# Inhibition of sphingosine-1-phosphate receptor 3 suppresses ATP-induced NLRP3 inflammasome activation in macrophages *via* TWIK2-mediated potassium efflux

**DOI:** 10.3389/fimmu.2023.1090202

**Published:** 2023-01-31

**Authors:** Yingqin Wang, Chen Wang, Qiaolan He, Guannan Chen, Jie Yu, Jing Cang, Ming Zhong

**Affiliations:** ^1^ Department of Anesthesiology, Zhongshan Hospital, Fudan University, Shanghai, China; ^2^ Department of Critical Care Medicine, Zhongshan Hospital, Fudan University, Shanghai, China; ^3^ Shanghai Key Laboratory of Lung Inflammation and Injury, Shanghai, China; ^4^ Shanghai Institute of Infectious Disease and Biosecurity, School of Public Health, Fudan University, Shanghai, China

**Keywords:** sepsis, NLRP3 inflammasome, sphingosine-1-phosphate receptor, TWIK2, macrophage

## Abstract

**Background:**

Inhibition of sphingosine kinase 1 (SphK1), which catalyzes bioactive lipid sphingosine-1–phosphate (S1P), attenuates NLRP3 inflammasome activation. S1P exerts most of its function by binding to S1P receptors (S1PR1-5). The roles of S1P receptors in NLRP3 inflammasome activation remain unclear.

**Materials and methods:**

The mRNA expressions of S1PRs in bone marrow-derived macrophages (BMDMs) were measured by real-time quantitative polymerase chain reaction (qPCR) assays. BMDMs were primed with LPS and stimulated with NLRP3 activators, including ATP, nigericin, and imiquimod. Interleukin-1β (IL-1β) in the cell culture supernatant was detected by enzyme-linked immunosorbent assay (ELISA). Intracellular potassium was labeled with a potassium indicator and was measured by confocal microscopy. Protein expression in whole-cell or plasma membrane fraction was measured by Western blot. Cecal ligation and puncture (CLP) was induced in C57BL/6J mice. Mortality, lung wet/dry ratio, NLRP3 activation, and bacterial loads were measured.

**Results:**

Macrophages expressed all five S1PRs in the resting state. The mRNA expression of S1PR3 was upregulated after lipopolysaccharide (LPS) stimulation. Inhibition of S1PR3 suppressed NLRP3 and pro-IL-1β in macrophages primed with LPS. Inhibition of S1PR3 attenuated ATP-induced NLRP3 inflammasome activation, enhanced nigericin-induced NLRP3 activation, and did not affect imiquimod-induced NLRP3 inflammasome activation. In addition, inhibition of S1PR3 suppressed ATP-induced intracellular potassium efflux. Inhibition of S1PR3 did not affect the mRNA or protein expression of TWIK2 in LPS-primed BMDMs. ATP stimulation induced TWIK2 expression in the plasma membrane of LPS-primed BMDMs, and inhibition of S1PR3 impeded the membrane expression of TWIK2 induced by ATP. Compared with CLP mice treated with vehicle, CLP mice treated with the S1PR3 antagonist, TY52156, had aggravated pulmonary edema, increased bacterial loads in the lung, liver, spleen, and blood, and a higher seven-day mortality rate.

**Conclusions:**

Inhibition of S1PR3 suppresses the expression of NLRP3 and pro-IL-1β during LPS priming, and attenuates ATP-induced NLRP3 inflammasome activation by impeding membrane trafficking of TWIK2 and potassium efflux. Although inhibition of S1PR3 decreases IL-1β maturation in the lungs, it leads to higher bacterial loads and mortality in CLP mice.

## Introduction

Sepsis is a critical clinical condition caused by infection. Globally, more than 19 million people are diagnosed with sepsis each year (1). Despite advances in supportive care for patients with sepsis, mortality remains over 25% (1). Currently, no specific treatment is available for sepsis. Sepsis is defined as a dysregulated host response to infection, and thus modulation of immune response is considered a reasonable approach to the treatment of sepsis.

Nucleotide-binding oligomerization domain-like receptor containing pyrin domain 3 (NLRP3) inflammasome is a multiprotein complex that triggers inflammatory responses in immune cells including macrophages (2). *Nlrp3* deficiency has been reported to protect mice from excessive pro-inflammatory cytokine storm and organ damage in inflammatory conditions including sepsis (3, 4). The activation of NLRP3 requires at least two signals (5). In the initial stage, the priming signals such as lipopolysaccharide (LPS) or tumor necrosis factor α (TNF-α) upregulate the transcription of NLRP3, interleukin-1β (IL-1β), and interleukin-18 (IL-18) in a nuclear factor κB (NF-κB)-dependent way (5). In addition, the priming signal licenses NLRP3 to rapidly respond to activating stimuli by post-translational modifications (6). Next, pathogen-associated molecular patterns (PAMPs) or danger-associated molecular patterns (DAMPs) such as adenosine triphosphate (ATP) act as the activation signal, which leads to the recruitment of the adaptor protein apoptosis-associated speck-like protein containing a caspase recruitment domain (ASC), never in mitosis A-related kinase 7 (NEK7) and pro-caspase-1 to NLRP3 (5). Then pro-caspase-1 is cleaved into bioactive caspase-1, which activates pro-IL-1β and pro-IL-18 into IL-1β and IL-18, respectively. Although reactive oxygen species production, metabolism disorder, mitochondrial dysfunction, lysosomal damage, and iron flux contribute contributes to NLRP3 activation, studies have shown that the decrease in intracellular potassium is an essential upstream event in NLRP3 activation induced by ATP and other DAMPs (7, 8). However, the mechanism of potassium efflux triggered by DAMPs remains poorly understood.

Sphingosine-1-phosphate (S1P) is a bioactive lipid that is generated from sphingosine by two key isoforms of sphingosine kinases, SphK1 and SphK2. S1P regulates diverse biologic processes by binding to five transmembrane sphingosine-1-phosphate receptors (S1PRs), S1PR1-S1PR5 (9). SphK1/S1P/S1PRs axis plays a key role in the pro-inflammatory response of macrophages and orchestrates the pathogenesis of inflammatory-related diseases such as inflammatory bowel disease, atherosclerosis, and infection (10). S1P is shown to stimulate NLRP3 inflammasome activation by acting as both priming and activation signals (11, 12). We have reported that inhibition of SphK1 improved the survival and lung vascular leakage in mice with cecal ligation and puncture (CLP)-induced sepsis by impeding NLRP3 inflammasome activation and subsequent IL-1β release from macrophages (11). However, the roles of S1PRs in NLRP3 inflammasome activation remain unclear. In this study, we explored whether the S1P receptors have a regulatory role in NLRP3 inflammasome activation and sepsis.

## Materials and methods

### Animals

Male C57BL/6 mice (10-to 12-week-old, 23-25g) were used in the experiments. All mice were housed in a specific pathogen-free facility with a 12:12 light: dark cycle and were free to food and water. Animal experimental protocols were approved by the Animal Care and Use Committee of the Zhongshan Hospital, Fudan University.

### Polymicrobial sepsis model and drug treatment

Polymicrobial sepsis was induced by cecal ligation and puncture (CLP). Mice were intraperitoneally anesthetized with pentobarbital (80 mg/kg). Under the aseptic condition, laparotomy was performed with a 1 cm cut in the lower abdomen. The cecum was ligated at the point of 1 cm from the distal end of the cecum followed by a puncture with a 16-gauge needle. A small amount of feces was exteriorized from the cecum and the cecum was returned to the peritoneal cavity. The sham surgery was performed as surgical control, where the same operations were conducted except for cecal ligation, puncture, and exteriorization of feces. TY52156 was resolved in dimethyl sulfoxide (DMSO) at a concentration of 20mg/ml. Twenty minutes before surgery, TY52156 (10 mg/kg) or DMSO was injected intraperitoneally. No antibiotics were given. An overdose of pentobarbital was used for the euthanasia of mice.

### Measurement of bacterial burden

Blood and tissues were aseptically harvested from mice at 24 h after CLP. Tissues were homogenized in sterile PBS at 4°C, and the homogenates were diluted 10-fold with sterile PBS. Then blood and tissue homogenates were plated on LB agar plates, which were incubated at 37°C for 16 to 24 h under aerobic conditions. Colony-forming units (CFUs) were calculated and log-transformed for statistical analysis.

### Measurement of wet/dry ratio

Left lung tissues harvested 24 h after CLP were weighed as the wet weight. Then, the lung tissues were dried in an oven at 60 °C for 48 h, the tissues were weighed again as the dry weight. The ratio of lung wet/dry weight was calculated.

### Cell culture and stimulation

Bone marrow cells were prepared from C57BL/6J mice, as previously described. In brief, bone marrow cells were differentiated into bone marrow-derived macrophages (BMDMs) for 6-7 days with DMEM/F12 medium (Hyclone, USA) supplemented with 20% L929 conditioned media and 10% heat-inactivate fetal bovine serum (FBS) (Biological Industries, Israel). The medium was replaced every 2-3 days. BMDMs were primed with 1 μg/ml LPS for 3 h in FBS free medium, with S1PR1 antagonist (W146, 3602, Tocris Bioscience, USA), S1PR2 antagonist (JTE-013, 10009458, Cayman, USA), S1PR3 antagonist (TY52156, 19119, Cayman, USA), S1PR4 antagonist (CYM 50358, 4679, Tocris Bioscience, UK), S1PR5 agonist (A971432, SML1744, Sigma-Aldrich, USA) or vehicle. After priming, BMDMs were stimulated with 3 mM ATP (A2383-5G, Sigma-Aldrich, USA), 10μM Nigericin (tlrl-nig, *In vivo*gen, USA), 25μg/mL imiquimod (tlrl-imq, *In vivo*gen, USA) for 30 min; 2μg/mL flagellin (tlrl-stfla, *In vivo*gen, USA) with 2.5 μl/ml of Lipofectamine 2000, 5μg/mL dsDNA μg (tlrl-patn, *In vivo*gen, USA) with 2.5 μl/ml of Lipofectamine 2000 for 6h. Cells were lysed for Western blot, and the supernatant was collected after stimulation.

### Real-time quantitative polymerase chain reaction assays

The total ribonucleic acid (RNA) was extracted from BMDMs using TRIzol reagent (Invitrogen, USA), and was converted to complementary deoxyribonucleic acid with the Reverse Transcription Master Mix (EZBioscience, USA). Quantitative real-time polymerase chain reaction (qPCR) was performed using SYBR Green qPCR Master Mix (EZBioscience, USA) according to the manufacturer’s protocols. Primers are listed in **Supplementary Table 1**

### Enzyme-linked immunosorbent assay

IL-1β in the supernatant was measured by Mouse IL-1 beta/IL-1F2 DuoSet enzyme-linked immunosorbent assay kit (R&D, DY401-05, USA) according to the manufacturer’s instructions.

### Western blot

Cells were lysed in Nonidet P-40 lysis buffer with phenylmethanesulfonyl fluoride and protease inhibitor. Boiled samples of cell lysate with loading buffer were electrophoresed and transferred onto a 0.2μm polyvinylidene fluoride membrane (Millipore, USA). Membranes were blocked with 5% nonfat dry milk for 1 h at room temperature followed by incubation with primary antibodies overnight at 4°C. Then the membranes were incubated with secondary antibodies for 1 h at room temperature and were detected with an enhanced chemical luminescence kit (Beyotime technology, China). Antibodies are shown in **Supplementary Table 2**.

### Measurement of intracellular potassium

Cells were loaded with a potassium indicator, ION Potassium Green-2 AM (ab142806, Abcam, UK) at 5μM for 30 min at room temperature, according to the manufacturer’s instructions. After two washes in warmed PBS, cells were imaged using an Olympus FV3000 inverted confocal microscope. The fluorescence signal between 530nm and 560 nm was recorded.

### Plasma membrane protein separation

The plasma membrane was isolated using Plasma Membrane Protein Extraction Kit (ab65400, Abcam, UK) according to the manufacturer’s instructions. One 10-cm plate of cells for each condition was scraped in phosphate-buffered saline (PBS). The cells were centrifugated at 3000 rpm for 5 minutes and washed once with ice-cold PBS. Then the cells were re-suspended in the Homogenize Buffer Mix in an ice-cold Dounce homogenizer and were homogenized 50 times. The homogenates were transferred to 1.5 ml microcentrifuge tubes and centrifuged at 700 g for 10 minutes at 4°C. The supernatants were transferred to new vials and centrifuged at 10,000 g for 30 min at 4°C to pellet total cellular membrane protein. Plasma membrane proteins were purified from the total cellular membrane protein with phase separation solutions and were pelleted and dissolved in 0.5% Triton X-100, followed by Western blot.

### Statistical analysis

A two-tailed unpaired Student’s t-test or Mann–Whitney U test was used to compare the two groups. Three or more groups were tested with the one-way analysis of variance (ANOVA) followed by Tukey’s *post hoc* test. The Kaplan–Meier plot with log-rank (Mantel-Cox) test was adopted to assess survival differences between groups. Grayscale or fluorescence intensity in image data was quantified with ImageJ (National Institutes of Health, USA). Statistical analyzes were performed in GraphPad Prism 9 (GraphPad Software Inc., USA). The RNA sequencing (RNAseq) data of healthy controls and septic patients on the Gene Expression Omnibus (GEO) database (GSE46955) was analyzed with an online GEO2R tool. The R scripts were got from GEO2R with some modifications to download and save the data. A two-tailed hypothesis test was used, and a p value less than 0.05 was considered statistically significant.

## Results

### Expression of S1PRs in macrophages in resting and inflammatory state

**Figure 1:**
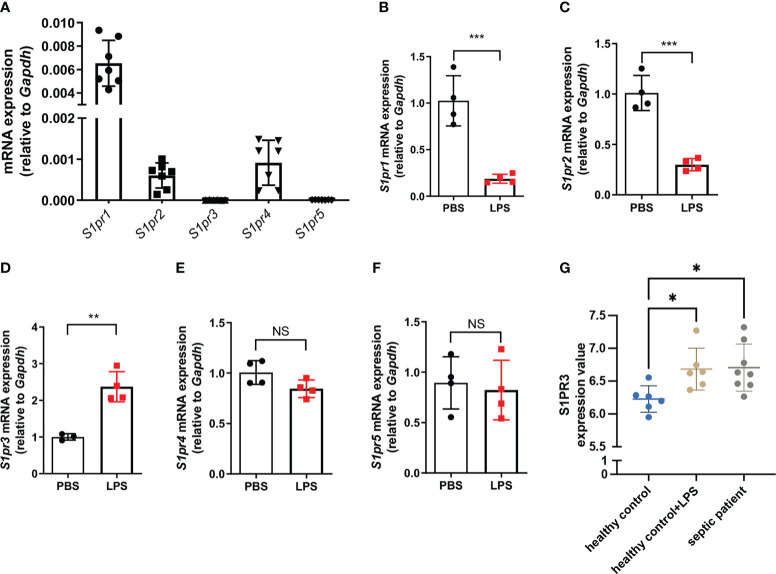
The expression of sphingosine-1-phosphate receptors in BMDMs. **(A)** Relative mRNA expression of S1PRs to GAPDH in BMDMs (n=7). **(B-F)** BMDMs were treated with LPS (1μg/mL) or PBS for 4h. The mRNA expressions of S1PRs in BMDMs were measured by RT-qPCR (n=4). **(G)** The mRNA expression values of S1PR3 in monocytes from healthy controls stimulated with or without LPS, and from septic patients were analyzed with data from the GEO database. Data are presented as mean ± SD. *p<0.05, **p < 0.01, ***p < 0.001, two-tailed t-test. NS, non-sense.

We first checked the relative mRNA expression of S1PR1-5 in BMDMs, and we found that BMDMs mainly expressed S1PR1, S1PR2, and S1PR4 in the resting state (**Figure 1A**). Only the expression of S1PR3 was upregulated in BMDMs after LPS stimulation (**Figures 1B-F**). Next, we analyzed the expressions of S1PR3 in monocytes of healthy volunteers and septic patients with the online RNAseq data. Five S1PRs were detected in human monocytes (**Supplementary Figure 1**). Human monocytes mainly expressed S1PR1 and S1PR4. The analysis showed that only the mRNA expression of S1PR3 was upregulated in human monocytes after LPS stimulation (**Supplementary Figure 1**). In addition, compared with healthy controls, septic patients had higher mRNA levels of S1PR3 in monocytes (**Figure 1G**).

### Inhibition of S1PR3 suppresses ATP-induced NLRP3 inflammasome activation in macrophages

**Figure 2:**
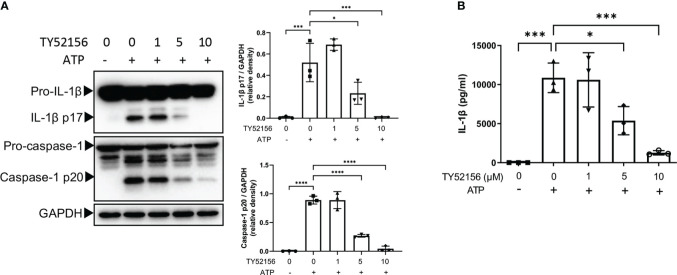
Inhibition of S1PR3 suppresses NLRP3 inflammasome activation induced by ATP. **(A, B)** BMDMs were primed with LPS (1μg/mL) and treated with TY52156 (0, 1, 5, 10μM) for 3.5h, followed by ATP (3mM) stimulation for 30 min. Pro-IL-1β, IL-1β p17, pro-caspase-1, and caspase-1 p20 in BMDMs were measured by Western blot (n= 3) **(A)**. IL-1β in the supernatant was measured by ELISA (n= 3) **(B)**. Values are presented as mean ± SD. *p < 0.05, ***p < 0.001, ****p < 0.0001, one-way ANOVA.

To determine the effects of S1PRs on NLRP3 activation, S1PR1-S1PR4 were inhibited with their respective antagonists during LPS priming followed by ATP stimulation in BMDMs, and S1PR5 was activated with its agonist during LPS priming because no commercial antagonist of S1PR5 was available (**Figure 2**; **Supplementary Figure 2**). Inhibition of S1PR3 significantly reduced both caspase-1 cleavage and IL-1β maturation in LPS-primed BMDMs after ATP stimulation (**Figure 2A**). Consistently, the level of IL-1β in the supernatant of BMDMs treated with LPS and the S1PR3 antagonist was lower than that of BMDMs treated with LPS (**Figure 2B**). Inhibition of S1PR2 also partially suppressed NLRP3 activation induced by ATP (**Supplementary Figure 2B**). Responses of BMDMs to ATP remained intact when treated with the S1PR1 antagonist or the S1PR4 antagonist (**Supplementary Figures 2A, C**). Activation of S1PR5 did not further potentiate IL-1β or caspase-1 activation in BMDMs after ATP stimulation (**Supplementary Figure 2D**). These results suggest that NLRP3 inflammasome activation induced by ATP is mainly limited by the suppression of S1PR3 signaling in BMDMs.

### Inhibition of S1PR3 suppresses the LPS priming signal in BMDMs

**Figure 3:**
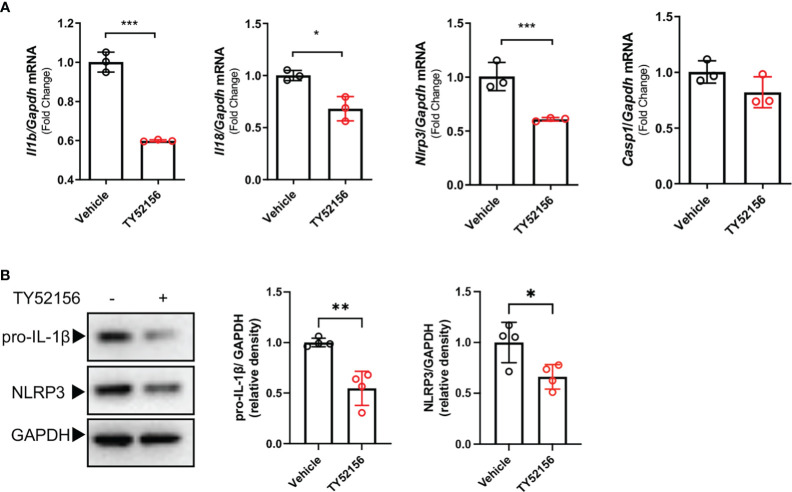
Inhibition of S1PR3 suppresses the LPS priming signal in BMDMs. **(A-B)** BMDMs treated with LPS (1μg/mL) for 4h with or without TY52156 (10 μM). The mRNA expressions of IL-1β, IL-18, NLRP3, and caspase-1 were measured by RT-qPCR **(A)**. The protein expression of pro-IL-1β and NLRP3 **(B)** were measured by Western blot (n=3). Values are presented as mean ± SD. *p < 0.05, **p < 0.01, ***p < 0.001, two-tailed t-test.

To understand the mechanism by which inhibition of S1PR3 suppressed NLRP3 inflammasome activation, we first examined the roles of S1PR3 in LPS priming. The results show that inhibition of S1PR3 during LPS priming downregulated mRNA expression of IL-1β, IL-18, NLRP3 (**Figure 3A**), and protein levels of pro-IL-1β and NLRP3 in BMDMs (**Figure 3B**). Inhibition of S1PR3 did not affect the mRNA and protein expression of pro-caspase-1(**Supplementary Figure 3**). These data suggest that inhibition of S1PR3 suppresses the expression of IL-1β and NLRP3 in macrophages during LPS priming.

### Inhibition of S1PR3 suppresses ATP-induced NLRP3 inflammasome activation *via* potassium efflux

**Figure 4:**
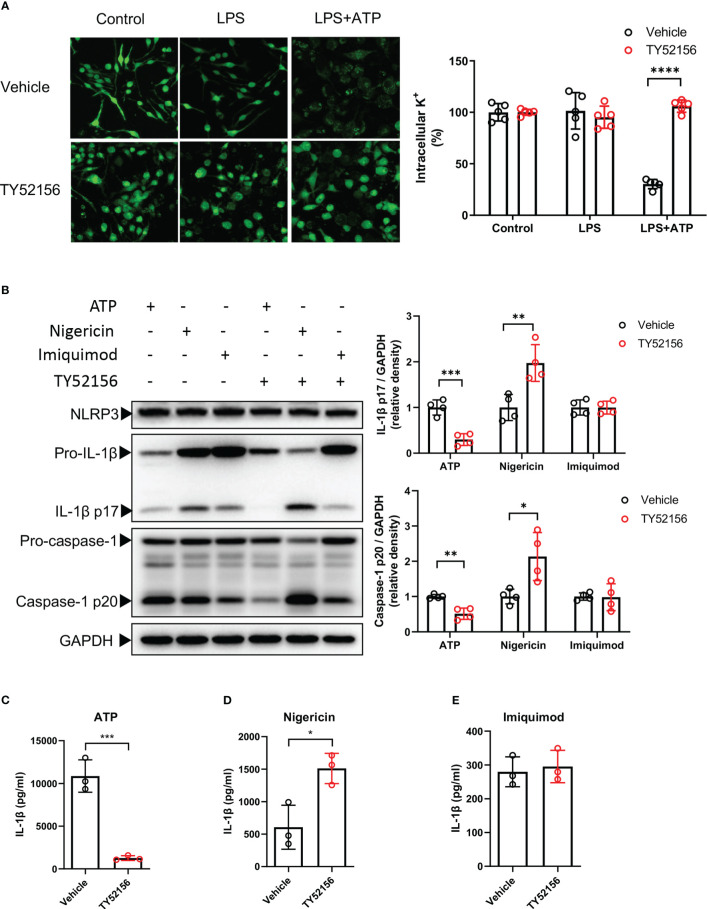
Inhibition of S1PR3 suppresses ATP-induced NLRP3 inflammasome activation *via* potassium efflux. **(A)** BMDMs were primed with LPS (1μg/mL) and treated with TY52156 (10μM) or vehicle for 3.5h, followed by ATP (3mM) stimulation for 30 min. Representative intracellular potassium fluorescence image of 5 fields from 3 independent experiments. Values are presented as mean ± SD. ****p < 0.0001, two-tailed t-test. **(B-E)** BMDMs were primed with LPS (1μg/mL) and treated with TY52156 (10μM) or vehicle for 3.5h, followed by ATP (3mM), nigericin (10μM), or imiquimod (25μg/mL) stimulation for 30 min. Pro-IL-1β, IL-1β p17, pro-caspase-1, and caspase-1 p20 in BMDMs were measured by Western blot (n= 4) **(B)**. IL-1β in the supernatant was measured by ELISA (n= 3) **(C-E)**. Values are presented as mean ± SD. *p < 0.05, **p < 0.01, ***p < 0.001, two-tailed t-test.

Next, we explored the roles of S1PR3 in NLRP3 activation in LPS-primed BMDMs. Given that potassium efflux is a common upstream of NLRP3 inflammasome induced by DAMPs including ATP (8), we tested whether S1PR3 can regulate potassium efflux induced by ATP. We found that inhibition of S1PR3 significantly reduced potassium efflux after ATP stimulation (**Figure 4A**). We further investigated the effects of inhibition of S1PR3 in NLRP3 inflammasome activation by nigericin, a bacterial potassium pore-forming toxin, which induces NLRP3 activation *via* directly mediating potassium efflux, and imiquimod, which activates NLRP3 inflammasome independent of potassium efflux (13). We found that inhibition of S1PR3 potentiated NLRP3 inflammasome activation induced by nigericin, but did not affect on that induced by imiquimod (**Figures 4B-E**). Furthermore, we found that inhibition of S1PR3 enhanced dsDNA-induced AIM2 inflammasome and salmonella infection-induced NLRC4 inflammasome activation, as indicated by enhanced caspase-1 cleavage (**Supplementary Figures 4A, B**). These results thus demonstrate a specific role of S1PR3 signaling in ATP-induced NLRP3 inflammasome activation by suppressing potassium efflux.

### Inhibition of S1PR3 suppresses ATP-induced potassium efflux through TWIK2 membrane trafficking

**Figure 5:**
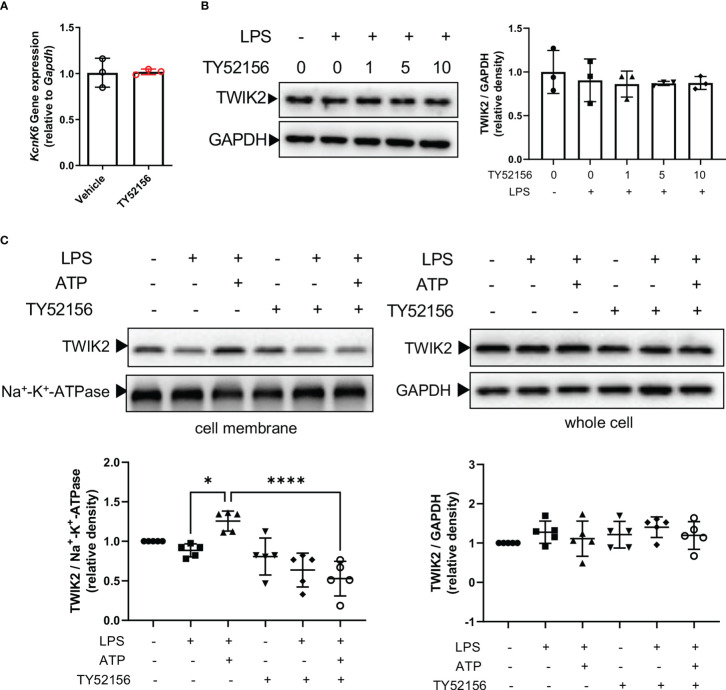
Inhibition of S1PR3 suppresses ATP-induced potassium efflux through TWIK2 membrane trafficking. **(A)** BMDMs were primed with LPS (1μg/mL) and treated with TY52156 (10μM) or vehicle for 4h. The mRNA expression of KcnK6, which codes TWIK2, was measured by RT-qPCR (n=3). Values are presented as mean ± SD. Data were analyzed with a two-tailed t-test. **(B)** BMDMs were primed with LPS (1μg/mL) and treated with TY52156 (0, 1, 5, 10μM) for 4h. The TWIK2 in BMDMs was measured by Western blot (n= 3). Values are presented as mean ± SD. Data were analyzed with one-way ANOVA. **(C)** BMDMs were primed with LPS (1μg/mL) and treated with TY52156 (10μM) or vehicle, followed by ATP (3mM) stimulation for 30 min. The TWIK2 in the plasma membrane and whole-cell were measured by Western bot (n= 5). Values are presented as mean ± SD. *p < 0.05, ****p < 0.0001, one-way ANOVA.

The evidence has shown that TWIK2-mediated potassium efflux is required for ATP-induced NLRP3 inflammasome activation (13). In macrophages, ATP binds to P2X7 receptors to activate NLRP3 inflammasome. Thus, we investigated the effect of S1PR3 on the expression of TWIK2 and P2X7 receptors in macrophages. Inhibition of S1PR3 did not affect the mRNA or protein expression of TWIK2 in BMDMs primed with LPS (**Figures 5A, B**). The mRNA levels of P2X7 receptors in macrophages were slightly increased after LPS stimulation, but the protein expression of P2X7 receptors was unchanged (**Supplementary Figures 5A, B**). Recent evidence has indicated that both TWIK2 and P2X7 receptors dynamically move between the plasma membrane and cytosol (14–17). We found that the expression of TWIK2 on the plasma membrane was induced by ATP stimulation in LPS-primed BMDMs (**Figure 5C**). Meanwhile, compared with the control BMDMs, less TWIK2 was expressed on the plasma membrane of S1PR3–inhibited BMDMs (**Figure 5C**). The protein expression of P2X7 receptors on the plasma membrane was unaffected by LPS, LPS plus ATP stimulation, or S1PR3 inhibition (**Supplementary Figure 5C**). These results suggest that inhibition of S1PR3 could suppress ATP-induced NLRP3 inflammasome activation by limiting TWIK2 membrane trafficking.

### Inhibition of S1PR3 increases mice mortality in polymicrobial sepsis

**Figure 6:**
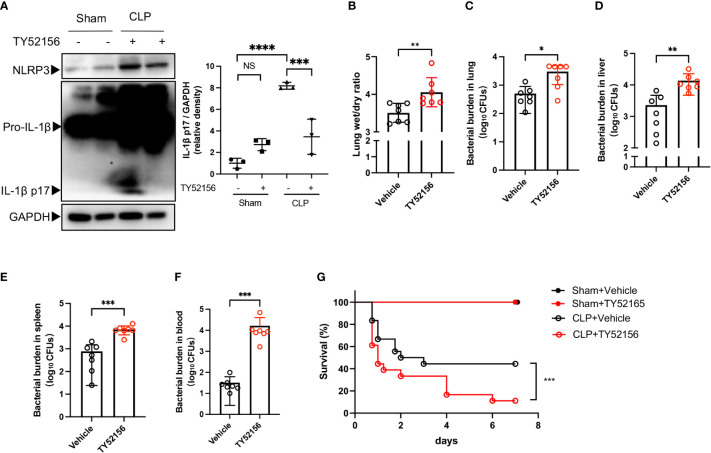
Inhibition of S1PR3 increases mortality and bacterial burden in sepsis. **(A)** Western blots of lung tissues from CLP mice treated with TY52156 or vehicle, and sham surgery control treated with TY52156 or vehicle (n=3 in each group) Values are presented as mean ± SD. ***p < 0.001, ****p < 0.0001, one-way ANOVA. **(B)** Lung wet/dry ratio of mice undergone CLP and then treated with TY52156 (10mg/kg) or vehicle (n=7 in each group). Values are presented as mean ± SD. **p < 0.01, two-tailed t-test. **(C-F)** Bacterial burden in the lung **(C)**, liver **(D)**, spleen **(E)**, and blood **(F)** of mice undergone CLP and then treated with TY52156 (10mg/kg) or vehicle (n=7 in each group). Values are presented as the mean of log10 CFU ± SD **(C-F)**. *p < 0.05, **p < 0.01, ***p < 0.001, Mann-Whitney test. **(G)** Kaplan Meier survival curves of mice undergone CLP or Sham surgery and then treated with TY52156 (10mg/kg) or vehicle (n=6 in Sham groups, n=18 in CLP groups). ***p < 0.001, log-rank test. NS, non-sense.

Next, we examined the role of S1PR3 in sepsis with the S1PR3 antagonist, TY52156. Twenty-four hours after CLP, mice in the S1PR3 inhibition group had lower levels of cleaved IL-1β in the lungs, compared with mice in the vehicle control group (**Figure 6A**). However, mice in the S1PR3 inhibition group had higher wet/dry ratios and heavier bacterial burdens in the lungs, livers, spleens, and blood than those in the vehicle control group (**Figures 6B-F**). The survival rate of CLP mice was 11.11% and 44.44% in the S1PR3 inhibition group and the vehicle control group (p < 0.001) (**Figure 6G**), respectively.

## Discussion

Activation of the inflammatory NLRP3 inflammasome is an important mechanism by which macrophages combat invading pathogens in the early stages of sepsis. Our data suggested that inhibition of S1PR3 suppressed the expression of NLRP3 and IL-1β during LPS priming and attenuated ATP-induced NLRP3 inflammasome activation by limiting TWIK2 membrane trafficking and subsequent potassium efflux (**Supplementary Figure 6**).

ATP is a common NLRP3 stimulus that was released in large quantities during sepsis, contributing to systemic inflammation and secondary organ damage (18, 19). Thus, we first tested the effects of S1PRs on NLRP3 inflammasome activation with ATP. Upon ATP stimulation, inhibition of S1PR3 significantly attenuated NLRP3 activation in LPS-primed macrophages. Notably, our results showed that inhibition of S1PR2 also partially hampered NLRP3 inflammasome activation in macrophages. A possibility is that the S1PR family members are functionally redundant. Indeed, it is reported that both S1PR2 and S1PR3 can activate the receptor-bound G-proteins G_αi_, G_αq_, and G_α12/13_ (20, 21). Evidence for the functional redundancy of S1PR2 and S1PR3 has also been described in modulating macrophage behaviors, such as motility in cholestatic liver injury (22), phenotype reprograming in mycobacteria infection (23), and phagocytosis in lung infection (24). Our results show that LPS stimulation only induced the expression of S1PR3 in both BMDMs and human monocytes, and that inhibition of S1PR3 attenuates NLRP3 inflammasome activation more efficiently than inhibition of S1PR2 in BMDMs. Therefore, we speculate that S1PR3 plays a major role in ATP-induced NLRP3 activation in macrophages under inflammatory conditions.

Potassium efflux is a main upstream event in NLRP3 inflammasome activation induced by various stimuli including ATP (13, 25). Our results indicate that inhibition of S1PR3 suppresses potassium efflux induced by ATP. Notably, inhibition of S1PR3 enhanced NLRP3 activation induced by nigericin, a potassium ionophore directly leading to potassium efflux (26, 27), and inhibition of S1PR3 had no effects on NLRP3 activation induced by imiquimod, which is independent of potassium efflux (28). Besides, inhibition of S1PR3 also enhanced AIM2 or NLRC4 inflammasome activation. These results suggest that the effects of S1PR3 on canonical inflammasome depend on the given stimulus and that inhibition of S1PR3 has an exclusive role in ATP-induced NLRP3 inflammasome activation *via* potassium efflux. Since ATP plays an essential role as an extracellular signaling molecule in the development of sepsis and septic organ failure (6), we focused on the mechanism of S1PR3 in ATP-induced NLRP3 inflammasome activation in our research. The mechanisms underlying the effects of S1PR3 on NLRP3 inflammasome activation induced by other stimuli need further investigation.

A recent study has identified TWIK2 as the potassium efflux channel required for ATP-induced NLRP3 inflammasome activation downstream of the ATP ligand, P2X7 receptors (13). However, the mechanism by which ATP enhanced potassium efflux *via* TWIK2 remains elusive. Our work here adds to the knowledge that ATP can stimulate potassium efflux by increasing the plasma membrane trafficking of TWIK2 without affecting the expression of P2X7 receptors. We also find that inhibition of S1PR3 limited the expression of TWIK2 on the cell membrane induced by ATP. Experimental data from Madin-Darby canine kidney (MDCK) cells show that exogenous expressed TWIK2 preferentially expresses in the Lamp1-positive lysosomal compartment, and functional relocates at the plasma membrane when its lysosomal targeting C terminal trafficking motifs are inactivated (17). Whether S1PR3 signaling engages with ATP-induced TWIK2 membrane trafficking by modulating this trafficking motif remains to be determined.

NLRP3 inflammasome activation is a double-edged sword in bacterial infection. On the one hand, a large number of inflammatory factors released after inflammasome activation can cause tissue damage; on the other hand, inflammasome activation and cell pyroptosis help to confine bacteria within macrophages, and the release of IL-1β facilitates the recruitment of neutrophils to clear bacteria (29). Previous studies have consistently demonstrated that *Nlrp3* deficiency protects mice from lethal sepsis, which is associated with lower levels of IL-1β (3, 4). In contrast, the benefit of *Nlrp3* deficiency in bacteria clearance in the septic mice remains controversial, which was possibly due to variations in experimental protocols (3, 4, 30, 31). Recent data has indicated that maintaining a certain amount of IL-1β prevented mice from death after sepsis in the long term (32). Clinical data shows that septic patients present an early impairment of the NLRP3 inflammasome is associated with a higher late-death rate (33). In our study, the antagonist of S1PR3 was used to investigate the effects of S1PR3 inhibition on CLP-induced septic mice. The dose of TY52156 we used, 10mg/kg, is also widely used in previously published studies (34, 35). In our results, this dose of TY52156 did not lead to the death of mice in the sham surgery group, which demonstrates that no significant toxicity of TY52156 is shown with this dose. Though inhibition of S1PR3 suppressed IL-1β activation in the lungs of septic mice, it also led to higher mortality and bacterial burdens, which were consistence with the results reported by Hou et al. in *S1pr3^-/-^* mice. Hou et al. have also reported that S1PR3 had a critical role in the bactericidal activity of macrophages (36). Compared with the healthy controls, the S1PR3 mRNA levels were higher in ICU controls and septic patients than that in healthy controls (36). However, the S1PR3 mRNA levels were negatively associated with the Sequential Organ Failure Assessment scores of the septic patients, and it was lower in septic non-survivors than that in septic survivors (36). Concerning the role of S1PR3 in promoting the bactericidal activity of macrophages, inhibition of S1PR3 can hamper both inflammation response and bacteria clearance, leading to poor outcomes.

## Conclusions

In conclusion, we report that suppression of S1PR3 has a specific inhibitory role in ATP-induced NLRP3 inflammasome activation in macrophages. Mechanically, inhibition of S1PR3 suppresses the expression of IL-1β and NLRP3 during LPS priming and attenuates NLRP3 inflammasome activation induced by ATP *via* downregulating membrane expression of TWIK2 and potassium efflux. Although inhibition of S1PR3 decreases IL-1β maturation in the lungs, it increases bacterial load and mortality of CLP mice.

## Data availability statement

Publicly available datasets were analyzed in this study. This data can be found here: https://www.ncbi.nlm.nih.gov/geo/query/acc.cgi?acc=GSE46955. Gene Expression Omnibus database (GSE46955).

## Ethics statement

The animal study was reviewed and approved by Zhongshan Hospital, Fudan University.

## Author contributions

Study conception and design: MZ, JC. *In vivo* experiment: YW, CW. *In vitro* experiment: YW. Collection and assembly of data, YW, CW, GC, JY. Data analysis and interpretation: YW, CW. Manuscript writing: YW. All authors contributed to the article and approved the submitted version.
