# Supplementary material for: Inhibition of sphingosine-1-phosphate receptor 3 suppresses ATP-induced NLRP3 inflammasome activation in macrophages via TWIK2-mediated potassium efflux
Source: Front Immunol. 2023 Jan 31;14:1090202. doi: 10.3389/fimmu.2023.1090202 (PMC9927198; doi:10.3389/fimmu.2023.1090202)
Supplement: Supplementary file 1 [file DataSheet_1.pdf]

Supplementary Material

1 Supplementary Figures

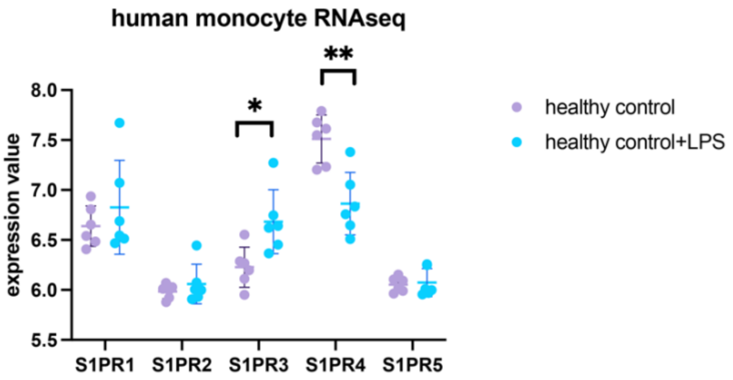

Supplementary Figure 1. The mRNA expression values of S1PRs in monocytes from healthy controls stimulated with or without LPS were analyzed with the data from the GEO database. *$p < 0.05$, **$p < 0.01$.

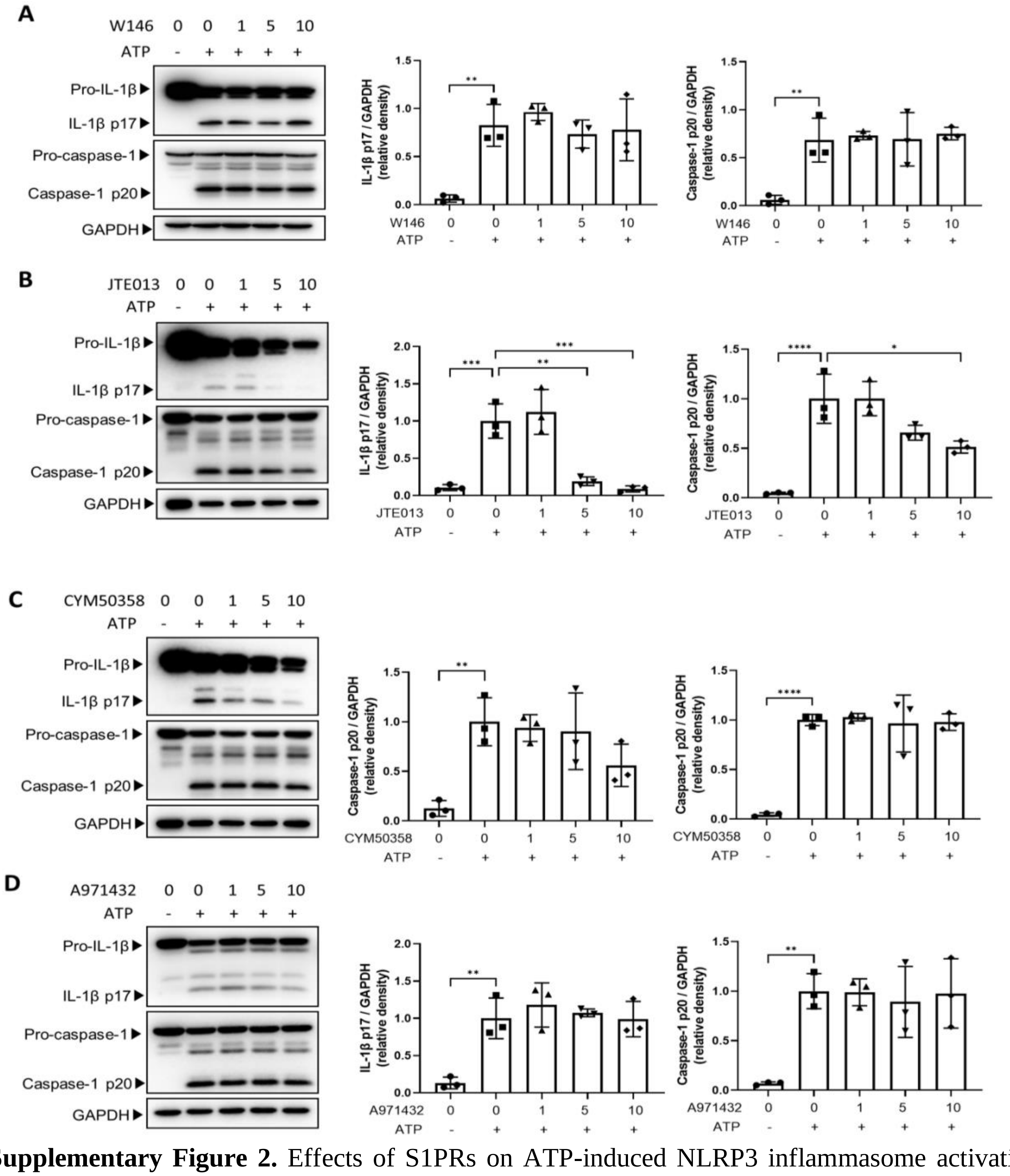

Supplementary Figure 2. Effects of S1PRs on ATP-induced NLRP3 inflammasome activation in macrophages. (A-D) BMDMs were primed with LPS (1μg/mL) and treated with S1PR1 antagonist (W146 at 0, 1, 5, 10μM) (A), S1PR2 antagonist (JTE013 at 0, 1, 5, 10μM) (B), S1PR4 antagonist (CYM50358 at 0, 1, 5, 10μM) (C), or S1PR5 agonist (A971432 at 0, 1, 5, 10μM) for 3.5h (D), followed by ATP (3mM) stimulation for 30 min. Pro-IL-1β, IL-1β p17, pro-caspase-1, and caspase-1 p20 in

BMDMs were measured by Western blot (n= 3). Values are presented as mean ± SD. *p < 0.05, **p < 0.01, ***p < 0.001, ****p < 0.0001, one-way ANOVA.

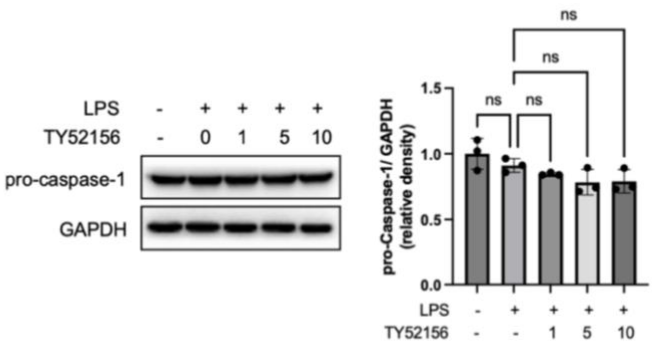

Supplementary Figure 3. Effects of inhibition of S1PR3 on the protein expression of pro-caspase-1 in LPS-primed BMDMs. BMDMs were primed with LPS (1μg/mL) and treated with TY52156 (0, 1, 5, 10μM) for 4h. Pro-caspase-1 in BMDMs were measured by Western blot (n= 3). Values are presented as mean ± SD, and tested with one-way ANOVA.

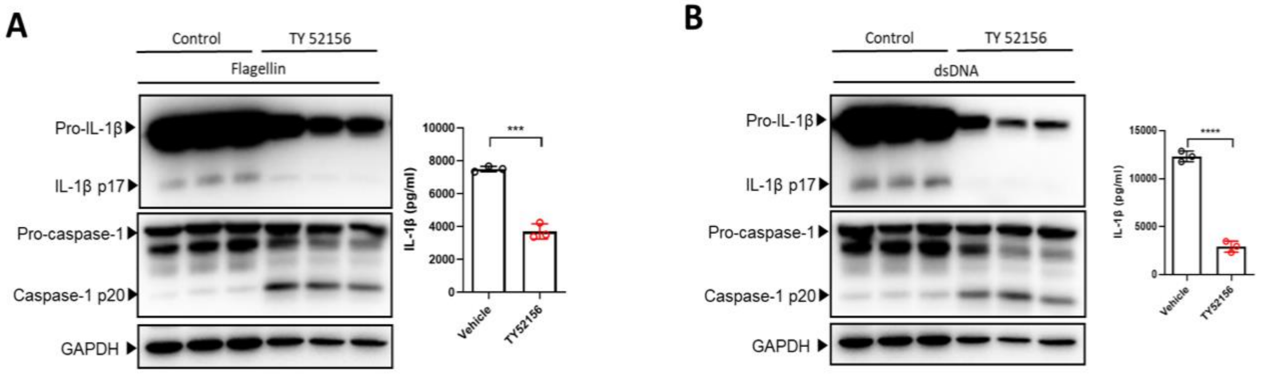

Supplementary Figure 4. Effects of inhibition of S1PR3 on NLRP4 and AIM2 inflammasome activation in macrophages. (A, B) BMDMs were primed with LPS (1μg/mL) and treated with TY52156 (10μM) for 3.5h, then stimulated with flagellin (2.5μg/mL) for 2h (A) or dsDNA (1μg/mL) for 6h (B). Pro-IL-1β, IL-1β p17, pro-caspase-1, and caspase-1 p20 in BMDMs were measured by Western blot (n= 3). IL-1β release from BMDMs were measured by ELISA (n= 3). Values are presented as mean ± SD. ***p < 0.001, ****p < 0.0001, two-tailed t-test.

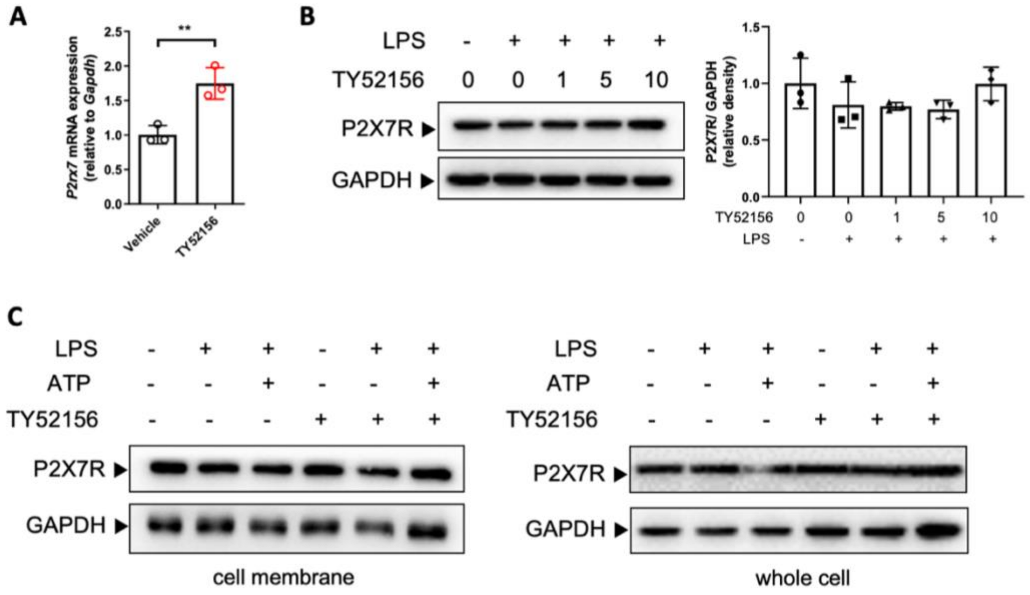

Supplementary Figure 5. Effects of inhibition of S1PR3 on P2X7 receptors in macrophages. (A) BMDMs were primed with LPS (1µg/mL) and treated with TY52156 (10µM) or vehicle for 4h. The mRNA expression of *P2xr7*, which codes the P2X7 receptor, was measured by RT-qPCR (n=3). Values are presented as mean ± SD. Data were analyzed with a two-tailed t test. (B) BMDMs were primed with LPS (1µg/mL) and treated with TY52156 (0, 1, 5, 10µM) for 4h. The P2X7 receptor in BMDMs was measured by Western blot (n= 3). Values are presented as mean ± SD. Data were analyzed with one-way ANOVA. (C) BMDMs were primed with LPS (1µg/mL) and treated with TY52156 (10µM) or vehicle, followed by ATP (3mM) stimulation for 30 min. The P2X7 receptor in the plasma membrane and whole-cell were measured by Western bot. ** p <0.01

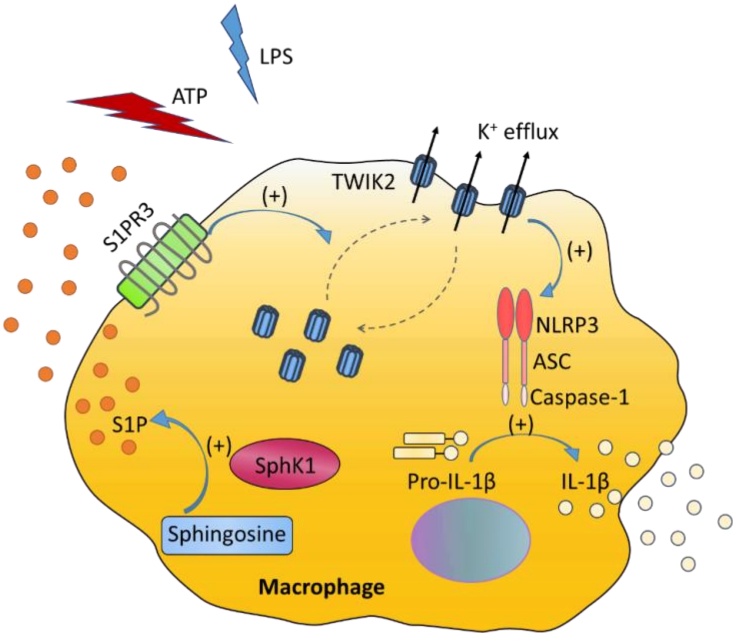

Supplementary Figure 6. S1PR3 signaling modulates ATP-induced NLRP3 inflammasome activation. Inhibition of S1PR3 suppresses ATP-induced NLRP3 inflammasome activation by limiting TWIK2 membrane trafficking and subsequent potassium efflux.

2 Supplementary Tables

Supplementary Table 1. Primers for real-time quantitative polymerase chain reaction assays

Gene	Forward sequence	Reverse sequence
S1pr1	GAAGATGAAACTACACAACGGG	CAGTAGAGGATGACGATGGAAA
S1pr2	TCTCTATGCTAAGCACTACGTG	GATGAAAACACCCAGTACGATG
S1pr3	TCTCCGGGAACATTACGATTAC	CCAAGTTGCCGATGAAAAAGTA
S1pr4	CTATGTGCTCTTTTGTGTGGTG	TTAGAACCAAAGATGTCAGCCA
S1pr5	CCTGCTTCGTACCCTTAGCG	GGCACGCGACATCCAGTAAT
Il1b	TCGCAGCAGCACATCAACAAGAG	TGCTCATGTCCTCATCCTGGAAGG
Casp1	AGAGGATTTCTTAACGGATGCA	TCACAAGACCAGGCATATTCTT
Nlrp3	ATTACCCGCCCGAGAAAGG	CATGAGTGTGGCTAGATCCAAG
KcnK6	TACACGACCCCACTGACAGAT	CAGTCAGAAGTAGCATGGTGAT
P2x7R	CTGGAACGATGTCTTGCAGTAT	CCCACTTGACGGTGCCATAAT
Gapdh	AAGAAGGTGGTGAAGCAGGCATC	CGGCATCGAAGGTGGAAGAGTG

Supplementary Table 2. Antibodies for Western blot

Antibody	Source	Catalog number
Anti-IL-1 beta Antibody	Abcam	ab9722
Anti-Caspase-1 Antibody	AdipoGen	AG-20B-0042-C100
Anti-NLRP3 Antibody	AdipoGen	AG-20B-0014-C100
Anti-TWIK2 Antibody	Santa Cruz	sc-376515
Anti-P2X7 Receptor Antibody	Alomone Labs	APR-004
GAPDH Monoclonal Antibody	ProteinTech	60004-1-Ig
Anti-mouse IgG, HRP-linked Antibody	CST	7076S
Anti-rabbit IgG, HRP-linked Antibody	CST	7074S
